# Cervical and systemic innate immunity predictors of HIV risk linked to genital herpes acquisition and time from HSV-2 seroconversion

**DOI:** 10.1136/sextrans-2022-055458

**Published:** 2022-09-14

**Authors:** Yashini Govender, Charles S Morrison, Pai-Lien Chen, Xiaoming Gao, Hidemi Yamamoto, Tsungai Chipato, Sharon Anderson, Robert Barbieri, Robert Salata, Gustavo F Doncel, Raina Nakova Fichorova

**Affiliations:** 1 Obstetrics, Gynecology and Reproductive Biology, Brigham and Women's Hospital, Boston, Massachusetts, USA; 2 Harvard Medical School, Boston, Massachusetts, USA; 3 Global Health and Population Research, FHI 360, Durham, North Carolina, USA; 4 Obstetrics and Gynecology, University of Zimbabwe, Harare, Zimbabwe; 5 Obstetrics and Gynecology, Eastern Virginia Medical School, Norfolk, Virginia, USA; 6 Medicine, Case Western Reserve University, Cleveland, Ohio, USA; 7 CONRAD, Arlington, Virginia, USA

**Keywords:** infection, inflammation, women, herpes genitalis, HIV

## Abstract

**Objectives:**

To examine innate immunity predictors of HIV-1 acquisition as biomarkers of HSV-2 risk and biological basis for epidemiologically established HIV-1 predisposition in HSV-2 infected women.

**Methods:**

We analysed longitudinal samples from HIV-1 negative visits of 1019 women before and after HSV-2 acquisition. We measured cervical and serum biomarkers of inflammation and immune activation previously linked to HIV-1 risk. Protein levels were Box-Cox transformed and ORs for HSV-2 acquisition were calculated based on top quartile or below/above median levels for all HSV-2 negative visits. Bivariate analysis determined the likelihood of HSV-2 acquisition by biomarker levels preceding infection. Linear mixed-effects models evaluated if biomarkers differed by HSV-2 status defined as negative, incident or established infections with an established infection cut-off starting at 6 months.

**Results:**

In the cervical compartment, two biomarkers of HIV-1 risk (low SLPI and high BD-2) also predicted HSV-2 acquisition. In addition, HSV-2 acquisition was associated with IL-1β, IL-6, IL-8, MIP-3α, ICAM-1 and VEGF when below median levels. Systemic immunity predictors of HSV-2 acquisition were high sCD14 and IL-6, with highest odds when concomitantly increased (OR=2.23, 1.49–3.35). Concomitant systemic and mucosal predictors of HSV-2 acquisition risk included (1) serum top quartile sCD14 with cervical low SLPI, VEGF and ICAM-1, or high BD-2; (2) serum high IL-6 with cervical low VEGF and ICAM-1, SLPI, IL-1β and IL-6; and (3) serum low C reactive protein with cervical high BD-2 (the only combination also predictive of HIV-1 acquisition). Most cervical biomarkers were decreased after HSV-2 acquisition compared with the HSV-2 negative visits, with incident infections associated with a larger number of suppressed cervical biomarkers and lower serum IL-6 levels compared with established infections.

**Conclusions:**

A combination of systemic immunoinflammatory and cervical immunosuppressed states predicts HSV-2 acquisition. A persistently suppressed innate immunity during incident HSV-2 infection may add to the increased HIV-1 susceptibility.

**WHAT IS ALREADY KNOWN ON THIS TOPIC**

- Genital herpes is a common risk factor for HIV acquisition, yet it is unknown if baseline patterns of mucosal and/or systemic innate immunity dysregulation are shared among and predispose to both viral infections.

**WHAT THIS STUDY ADDS**

- This study identifies for the first time both shared and divergent innate immunity predictors of HSV-2 and HIV-1 infection. It is first to show differences in both cervical and systemic innate immunity mediators that may underlie the higher risk of HIV acquisition in recent compared with established HSV-2 infections.

**HOW THIS STUDY MIGHT AFFECT RESEARCH, PRACTICE OR POLICY**

- By identifying molecular predictors of HSV-2 risk, this study provides targets and clinical safety endpoints for the development of preventive products.

## Introduction

The significance of herpes simplex virus-2 (HSV-2) as a risk factor for HIV is driven by the high HSV-2 prevalence worldwide and in the Sub-Saharan HIV epicentre (estimated 39.3%−83.3% in South African women).^1^ Women and men infected with HSV-2 have estimated 3-fold higher HIV acquisition.^2^ A 3.2-fold and 4.6-fold increased risk of HIV acquisition was associated with HSV-2 among 4500 Ugandan and Zimbabwean women, respectively.^3^ The same study found greater HIV acquisition with recent (within 3–21 months from a negative test) compared with prevalent HSV-2 infections in both Uganda (4.6-fold vs 2.8-fold) and Zimbabwe (8.6-fold vs 4.4-fold). These data are supported by findings of greater frequency and severity of clinically active herpes episodes after recent HSV-2 infections,^2^ which cause breaches in the epithelial layer and influx of activated immune cells, providing HIV with access to target cells.^4 5^ The possibility of HSV-2-initiated clinical or subclinical mucosal inflammation and innate immunity imbalance^5 6^ has been proposed as additional mechanism linking these viral infections but needed validation in a large clinical study.

Biomarkers of innate immunity predicted HIV-1 risk in the Hormonal Contraceptive and Risk of HIV (HC-HIV) cohort—one of the largest prospective studies examining the role of HC in HIV acquisition among African women.^7 8^ Both mucosal and systemic immunity imbalances contributed to the HIV risk^7^ and aberrant cervical immunity preceded other sexually transmitted infections including HSV-2.^8^ It remained unknown whether the imbalance predisposing to HSV-2 is limited to the cervical compartment or extends to the systemic circulation and whether HSV-2 in turn may alter both mucosal and peripheral innate immunity to contribute to HIV-1 risk. We hypothesised that (1) aberrant systemic immunity concomitantly with altered cervical immunity precedes and predisposes to HSV-2 infection, (2) HSV-2 infection changes cervical and systemic innate immunity, and (3) these changes may depend on the duration of HSV-2 infection which may add to the biological explanation of the greater HIV acquisition risk in incident vs established HSV-2 infections. To address these gaps, we analysed longitudinal specimens collected by the HC-HIV study and designed analysis models based on (1) HSV-2 status by visit (negative, incident or established) and (2) HSV-2 status by participant (remained negative or HSV-2 seroconverted).

## Methods

### Study population and visits

Biospecimens from 5193 HIV-negative visits by 1275 women were available from the HC-HIV study. Infections within 6 months (180 days) after first becoming HSV-2 seropositive were considered incident while those >6 months after a visit with confirmed positive seroconversion were considered established. The biological rationale for choosing the 6-month cut-off was based on observations of more HSV shedding within the first 6 months after acquisition^9^ and decreased clinical reactivation over time^10^ expected to be associated with changes in immunity. To investigate our hypotheses, we defined two population models within our cohort. Model 1 was based on HSV-2 infection status at the study visits grouped into (1) HSV-2 negative, (2) incident HSV-2 and (3) established HSV-2. Model 2 was defined by HSV-2 acquisition status as (1) remaining negative throughout the study with a minimum of two HSV-seronegative visits and (2) seroconverted during the study. The median number of visits was 2 for women remaining HSV-2 negative, and 3 for women with incident and established HSV-2 infections.

To accurately categorise visits as HSV-2 negative, incident or established infection by the aforementioned criteria, we excluded (1) all HSV-2 negative less than 12 weeks apart from a prior or follow-up serology test (including baseline and <12 weeks from study exit), (2) all visits preceding seroconversion by <12 weeks thus eliminating uncertainty of whether a woman could have been infected but not yet developed detectable antibodies and (3) all visits seropositive at baseline or <6 months from baseline as in both cases categorisation as incident or established would not be certain. We identified 3116 visits from 1019 women (413 Ugandan and 606 Zimbabwean) who met our inclusion criteria and infection definitions.

### Laboratory diagnosis

HSV-2 status was determined by a type-specific serological IgG antibody assay (Focus Technologies, Cypress, California, USA) as described.^3^ *C. trachomatis* (CT) and *N. gonorrhoeae* (NG) were diagnosed by PCR; *T. vaginalis* (TV) and *Candida* by wet mount. Abnormal microbiota and bacterial vaginosis (BV) were assessed by Nugent scoring. HIV status was determined by ELISA and confirmed by PCR.

### Biomarker measurement

Cervical swabs were processed as described.^11^ Ten cervical biomarkers (interleukin (IL)−1β, IL-6, IL-8, IL-1 receptor antagonist (IL-1RA), RANTES, MIP-3α, VEGF, soluble leukocyte protease inhibitor (SLPI), beta defensin (BD)2 and intercellular adhesion molecule (ICAM)-1) and four serum biomarkers (IL-6, IL-7, C reactive protein (CRP) and soluble (s)CD14) were measured as described in detail.^7 11^These biomarkers and their combinations were chosen for their proven role in vaginal innate immunity, reliable detection and established role as predictors of other STIs and HIV-1 acquisition in the Ugandan and Zimbabwean cohorts.^11–13^

### Statistical analyses

We compared participants’ baseline characteristics by HSV-2 status (HSV-2 negative visits, HSV-2 incident visits and HSV-2 established visits) using joint χ^2^ tests via the generalised estimating equation approach and the Freeman-Halton test if numbers of visits were less than 5. Participants providing at least one biospecimen and participants with both unpaired and paired bio (had both cervical and serum biomarkers) were included. Because immunity biomarker levels do not follow Gaussian distribution, concentrations were normalised using Box-Cox power transformation. Serum samples were analysed in one batch. Cervical biospecimens were analysed in two assay batches 4 years apart and data were harmonised for batch variation as previously described.^7^

Generalised linear mixed-effects models evaluated if levels of systemic and cervical immune mediators differ among HSV-2 negative, incident or established visits and adjusted for covariates. Bivariate analysis determined the OR of HSV-2 seroconversion with individual/grouped biomarker levels activated or suppressed at the quarterly visit prior to the incident visit. The categorisation into high (activated) or low (suppressed) and grouped analysis replicated cut-off rationale and biomarker combinations previously examined as predictors of HIV-1 acquisition.^7^ A Spearman rank-order test showed weak correlations between and within anatomical compartments (online supplemental table 1) supporting the choice of assessing categorically individual and combined biomarkers based on the biological rationale described previously. P values <0.05 were considered significant. Statistical analyses were performed using SAS V.9.4 (SAS Institute, Cary, NC, USA).

10.1136/sextrans-2022-055458.supp1 Supplementary data

## Results

This analysis included data from 3116 HIV-1 negative visits from Zimbabwe (64%) and Uganda (36%) of which 1505 were HSV-2 negative (48.3%), 633 were HSV-2 incident (20.3%) and 978 were HSV-2 established (31.4%) visits (online supplemental table 2). Most visits (59%) were from women 18–24 years old. Visits were equally distributed by DMPA, COCs and no-hormonal method use. Few visits were from pregnant (8%) or breastfeeding women (15%). Almost a third of visits (28%) were from women with BV and 11% had candidiasis while chlamydia (2%), gonorrhoea (2%) and trichomoniasis (3%) were rare.

HSV-2 negative visits were more likely from Zimbabwe (66%) and from younger women (66%). The majority of HSV-2 negative visits were contributed by women who remained HSV-2 negative (77%) while the remainder were collected from HSV-2 seroconverters at least 3 months prior to the incident visit. Incident visits were more likely from younger women (61%) while established visits more likely from older women (52%), from Zimbabwe (70%) and with BV (30%) (online supplemental table 2).

### Cervical and systemic biomarkers preceding and predicting HSV-2 acquisition

To determine whether systemic immunity may contribute to the risk of HSV-2 acquisition, either independently or in conjunction with altered cervical immunity, we first measured individual cervical and systemic biomarkers at the 3-month visit preceding HSV-2 seroconversion. Then we assessed whether inflammatory or immunosuppressive status concomitant at both systemic and cervical sites predisposed to HSV-2.

#### Individually altered biomarkers

In bivariate modelling, 5 of the 10 cervical and 2 of the 4 systemic biomarkers were individually associated with subsequent HSV-2 acquisition. Higher odds were found with cervical high BD-2 (OR=1.45, 95% CI 1.09 to 1.93, p=0.01), low SLPI (OR=1.50, 95% CI 1.13 to 2.00, p<0.01) or low ICAM-1 (OR=1.41, 95% CI 1.06 to 1.88, p=0.02). Lower odds of HSV-2 acquisition were found with cervical high IL-6 or high MIP-3α (OR=0.75, 95% CI 0.56 to 1.00, p<0.05). Systemic markers associated with subsequent HSV-2 acquisition included high sCD14 (OR=1.93, 95% CI 1.34 to 2.78, p<0.001) and IL-6 (OR=1.53, 95% CI 1.09 to 2.14, p=0.01) (table 1).

**Table 1:** Estimated ORs and 95% CIs for acquiring HSV-2 within the next 3 months with levels of individual and grouped biomarkers measured at the HSV-2 negative visit preceding seroconversion and compared with the visits contributed by the control women who never acquired HSV-2

Category	Biomarker	Samples ↑ or ↓*/sample used†	OR‡ (95% CI)	P value
**Cervical**	BD2↑	677/1365	**1.45 (1.09 to 1.93)**	**0.012**
	SLPI↓	661/1366	**1.50 (1.13 to 2.00)**	**0.006**
	IL-1RA↓	669/1366	1.18 (0.89 to 1.57)	0.256
	IL-1β↑	697/1366	0.83 (0.62 to 1.10)	0.200
	IL-6↑	696/1366	**0.75 (0.56 to 1.00)**	**0.048**
	IL-8↑	716/1366	0.78 (0.58 to 1.03)	0.082
	MIP-3α↑	696/1366	**0.75 (0.56 to 1.00)**	**0.048**
	RANTES↑	690/1366	1.07 (0.81 to 1.43)	0.628
	ICAM-1↓	672/1366	**1.41 (1.06 to 1.88)**	**0.018**
	VEGF↓	648/1366	1.07 (0.81 to 1.43)	0.630
	IL-1β↑, IL-6↑	509/1366	**0.58 (0.42 to 0.80)**	**0.001**
	IL-8↑, MIP-3α↑	535/1366	**0.68 (0.50 to 0.92)**	**0.012**
	ICAM-1↓, VEGF↓	366/1366	**1.47 (1.08 to 2.00)**	**0.013**
**Serum**	sCD14↑	192/787	**1.93 (1.34 to 2.78)**	**<0.001**
	CRP↓	391/787	1.14 (0.82 to 1.59)	0.439
	IL-6↑	391/787	**1.53 (1.09 to 2.14)**	**0.014**
	IL-7↑	392/787	1.17 (0.84 to 1.63)	0.366
	IL-6↑, IL-7↑	231/787	1.32 (0.92 to 1.88)	0.130
	sCD14↑, IL-6↑	130/787	**2.23 (1.49 to 3.35)**	**<0.001**
	sCD14↑, IL-6↑, IL-7↑	80/787	**1.73 (1.05 to 2.86)**	**0.033**

*↑ indicates levels above and ↓ below median of all 1505 HSV-2 negative study visits for all biomarkers except sCD14 where ↑ indicates levels above top quartile.

‡ORs and 95% CIs estimate likelihood of subsequent HSV-2 seroconversion with biomarker levels at the HSV-2 negative visit either below or above median/top quartile.

Bold values indicate significance at p<0.05.

BD2, Beta defensin 2; CD, cluster of differentiation; CI, Confidence interval; CRP, C reactive protein; HSV, herpes simplex virus; ICAM, Intercellular Adhesion Molecule; IL, interleukin; IL-1RA, interleukin 1 receptor antagonist; MIP, macrophage inflammatory protein; OR, Odds ratio; RANTES, Regulated upon Activation, Normal T Cell Expressed and Presumably Secreted; SLPI, secretory leukocyte protease inhibitor; VEGF, vascular endothelial growth factor .

#### Concomitantly altered biomarkers within each anatomical compartment

We assessed the predictive value of combined biomarkers within the same anatomical compartment. In cervical secretions, concomitant high IL-1β and IL-6 (OR=0.58, 95% CI 0.42 to 0.80, p=0.001) or IL-8 and MIP-3α (OR=0.68, 95% CI 0.50 to 0.92, p=0.01) indicated decreased risk while low ICAM-1 and VEGF (OR=1.47, 95% CI 1.08 to 2.00, p=0.01) increased risk of HSV-2 acquisition. Within the systemic circulation, increased HSV-2 acquisition risk was associated with the combinations of high sCD14 and IL-6 (OR=2.23, 95% CI 1.49 to 3.35, p<0.001) or high sCD14, IL-6 and IL-7 (OR=1.73, 95% CI 1.05 to 2.86, p=0.03) (table 1).

#### Combined cervical and systemic biomarkers

**Figure 1:**
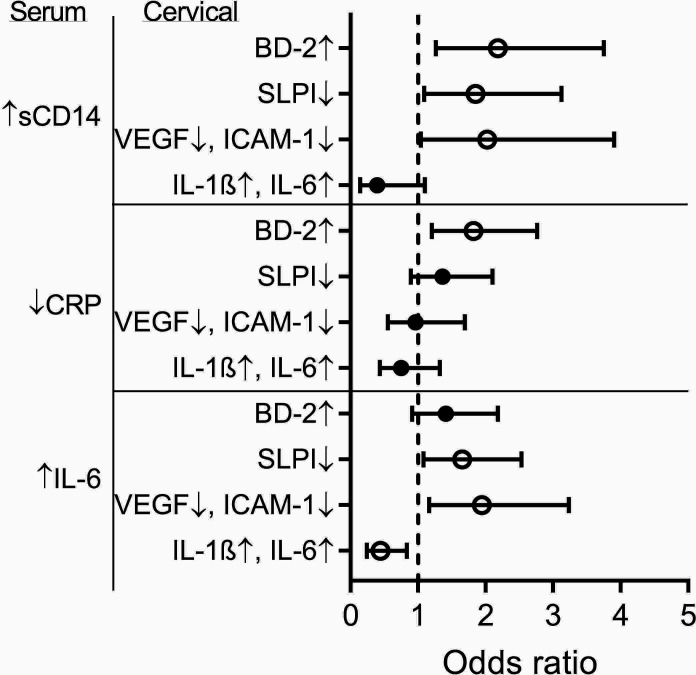
Forest plot of estimated ORs of HSV-2 acquisition for levels of concomitantly imbalanced systemic and cervical immunity assessed 3 months prior to the HSV-2 seroconversion visit. The ORs with 95% CIs are denoted by open circles for significant (p<0.05) and closed circles for non-significant p values. ↑ indicates levels above and ↓ below the median of all HSV-2 negative study visits for all biomarkers except sCD14 where ↑ indicates levels above top quartile. Bivariate analysis compared HSV-2 seroconverters at the visit prior to the incident visit to HSV-2 negative visits from individuals remaining HSV-2 negative throughout.

We found additional significant predictive patterns when we combined concomitantly aberrant cervical and systemic biomarkers. High systemic sCD14 in combination with either low cervical SLPI (OR=1.85, 95% CI 1.09 to 3.12, p=0.02) or high cervical BD-2 (OR=2.18, 95% CI 1.26 to 3.75, p=0.005) or low cervical VEGF and ICAM-1 (OR=2.02, 95% CI 1.04 to 3.90, p=0.04) conveyed higher odds of HSV-2 acquisition at the subsequent visit. High systemic IL-6 in combination with low cervical VEGF and ICAM-1 (OR=1.94, 95% CI 1.16 to 3.23, p=0.01) or with low cervical SLPI (OR=1.65, 95% CI 1.08 to 2.53, p=0.02) was associated with increased HSV-2 acquisition. High systemic IL-6 in combination with high cervical IL-1β and IL-6 was associated with decreased HSV-2 acquisition (OR=0.44, 95% CI 0.24 to 0.83, p=0.01). Low systemic CRP in combination with high cervical BD-2 (OR=1.82, 95% CI 1.20 to 2.76, p=0.005) was associated with increased HSV-2 acquisition (figure 1 and online supplemental table 3).

**Figure 2:**
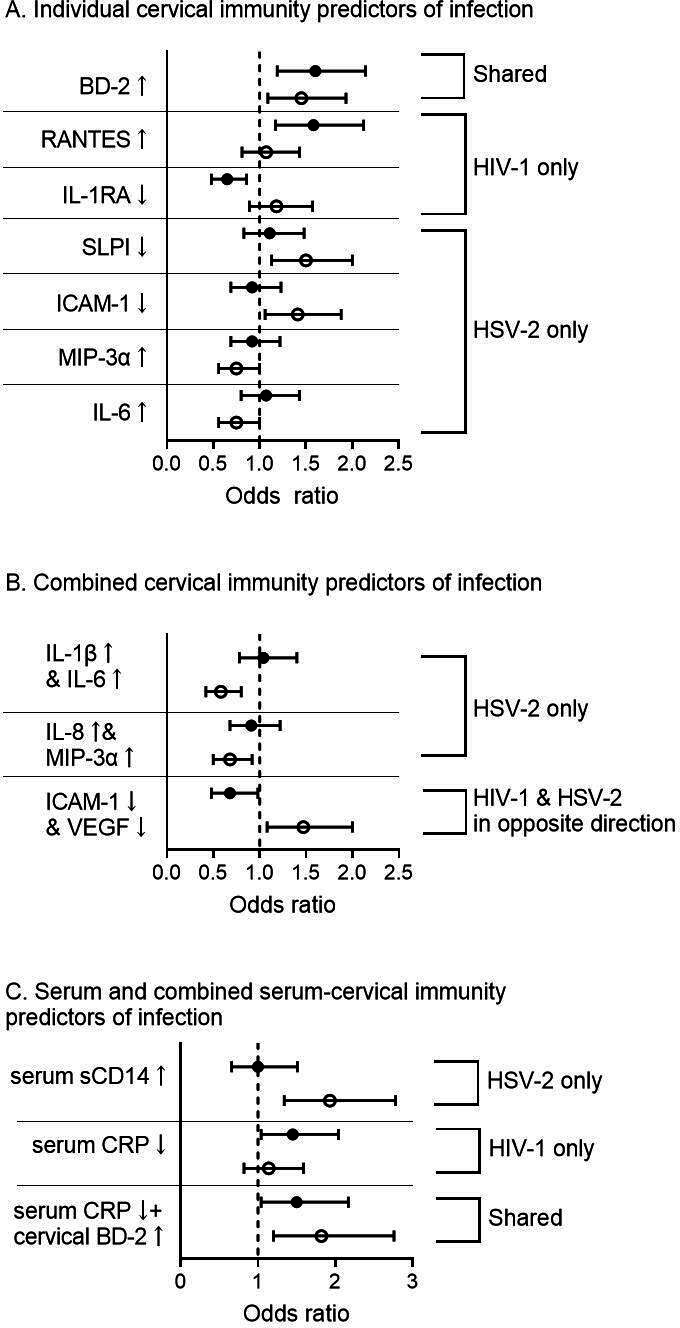
Forest plot of estimated ORs and 95% CIs of HSV-2 and HIV-1 acquisition for levels of imbalanced systemic and cervical immunity assessed 3 months prior to the HSV-2 or HIV-1 seroconversion visit. Only biomarkers or biomarker combinations significantly predicting either one or both viral infections are shown (p<0.05). The OR and 95% CI for HIV predictors are marked with closed circled and were taken from analyses previously described in Morrison *et al*.^7^ The OR and CI for the HSV-2 predictors are denoted with open circles. ↑ indicates levels above and ↓ below the median for all biomarkers except sCD14 where ↑ indicates levels above top quartile. Bivariate analysis compared seroconverters at the visit prior to seroconversion to all visits from individuals remaining negative for either infection.

We next contrasted the HSV-2 to HIV-1 immune predictors previously identified in the same cohort by the same statistical method.^7 8^ In the cervical compartment (figure 2A), high hBD-2 predicted both HSV-2 and HIV-1 acquisition, while high RANTES or low IL-1RA predicted HIV-1 acquisition only and low SLPI, ICAM-1 or high MIP-3α or IL-6 predicted HSV-2 acquisition. Different patterns of grouped proinflammatory (IL-1β and IL-6 or IL-8 and MIP-3α) or suppressed antiviral (ICAM-1 and VEGF) cervical immunity preceded and predicted HIV-1 and HSV-2 seroconversion (figure 2B). At the systemic level, low CRP predicted HIV-1 while high sCD14 predicted HSV-2 acquisition (figure 2C). A pattern of low serum CRP combined with high cervical BD-2 was a shared predictor of HIV-1 and HSV-2 acquisition (figure 2C).

### Differences in cervical and systemic immunity by HSV-2 incident and established infection status

Analysis adjusted for relevant covariates confirmed our hypotheses that HSV-2 infection changes cervical and systemic innate immunity, and that incident and established infections differentially influence these changes. We found lower levels of 7/10 cervical biomarkers in HSV-2 incident visits compared with HSV-2 negative visits contributed by all women throughout the study. Significant differences included lower SLPI, IL-1RA, IL-1β, IL-6, IL-8, MIP-3α and VEGF (table 2). In contrast, HSV-2 established visits had lower levels of 5/10 cervical mediators including SLPI, IL-6, IL-8 and VEGF and adding significantly lower BD-2 (p<0.01) compared with the HSV-2 negative visits. In a direct comparison of incident versus established visits, women with incident infections had lower levels of cervical IL-1β and MIP-3α, but also lower systemic IL-6 compared with women with established HSV-2 infections (p<0.01).

**Table 2:** Differences in levels* of cervical and systemic biomarkers by HSV-2 infection status adjusted for covariates

		HSV-2 status at the visit		
Cervical biomarker†	No of specimens	Incident (n=491) vs negative (n=1203)	Established (n=838) vs negative (n=1203)	Incident (n=491) vs established (n=838)
BD2	2530		↓**	
SLPI	2532	↓*	↓**	
IL-1RA	2532	↓**		
IL-1β	2532	↓***		↓**
IL-6	2532	↓***	↓***	
IL-8	2532	↓**	↓*	
MIP-3α	2532	↓***		↓**
RANTES	2532			
ICAM-1	2532			
VEGF	2532	↓**	↓*	
**Serum biomarker‡**	**No of specimens**	**Incident (n=390) vs negative (n=646)**	**Established (n=361) vs negative (n=646)**	**Incident (n=390) vs established (n=361)**
sCD14	1397			
CRP	1397			
IL-6	1396			↓**
IL-7	1396			

*↑ indicates significantly higher levels and ↓ indicates significant lower levels of these mediators compared with an HSV-2 negative reference (including all negative visits contributed by all women with information on adjusted variables listed in the models below) or the established HSV-2 reference ***p<0.001; **p<0.01; *p<0.05.

†The generalised linear mixed-effects models of differences by cervical biomarkers are adjusted for country, age, 5-level hormonal variable (pregnant/breastfeeding/majority COC/majority DMPA/majority NH), unprotected sex acts, current sexually transmitted or reproductive tract infections (STIs/RTIs including BV, CT, NG and TV), vaginal drying and cleaning practices.

‡The generalised linear mixed-effects models of differences by serum biomarkers are adjusted for country, age, 5-level hormonal variable (pregnant/breastfeeding/majority COC/majority DMPA/majority NH), and current sexually transmitted or reproductive tract infections (STIs/RTIs).

BD2, beta defensin 2; CD, cluster of differentiation; CRP, C reactive protein; HSV, hepes simplex virus; ICAM, intercellular adhesion molecule; IL, iterleukin; IL-1RA, interleukin 1 receptor antagonist; MIP, macrophage inflammatory protein; RANTES, Regulated upon Activation, Normal T Cell Expressed and Presumably Secreted; SLPI, secretory leukocyte protease inhibitor; VEGF, vascular endothelial growth factor.

## Discussion

This study provides evidence that while imbalances in some cervical innate immunity mediators may precede and predict both HIV-1 and HSV-2 infection, the two viral infections can be distinguished by antecedent patterns derived from both the mucosal and peripheral immune compartments, suggesting both common and divergent mechanisms of antiviral defence. Moreover, we show that HSV-2 infection not only changes innate immunity parameters previously associated with HIV risk but that the changes occurring within 6 months of HSV-2 infection differ from those observed later. These differences shed light on potential mechanisms, underlying epidemiological findings of a greater HIV acquisition risk with more recent HSV-2 infection.

To our knowledge, this study is the first to implicate systemic immunity and combined mucosal-systemic patterns in HSV-2 risk. We had previously shown that top quartile levels of cervical IL-6, SLPI and ICAM-1 decrease the likelihood of HSV-2 incidence.^8^ Consistent with and expanding those findings we now report that a generalised immunosuppressive state in the cervical compartment, characterised by SLPI↓, IL-6↓ (alone or combined with IL-1β↓), MIP-3α↓ (alone or combined with IL-8↓) or ICAM-1↓ (alone or combined with VEGF), predicts incident HSV-2 infection. BD-2 was the only upregulated marker preceding HSV-2 acquisition. In contrast to the permissive immunosuppressed status at the cervix, the systemic circulation displayed immunoinflammatory activation (serum sCD14↑, IL-6↑ or concomitant increase of both) to be predictive of HSV-2 acquisition.

SLPI is an antimicrobial protein with anti-inflammatory properties^14^ and has been shown to inhibit both HSV-2^15^ and HIV-1^16^ infection, which is consistent with our results of cervical SLPI↓ being predictive of both HSV-2 (reported here) and HIV-1 acquisition (reported previously).^7^ BD-2 is also an antimicrobial peptide and is part of the protective host responses to infection of the vaginal mucosa.^17^ However, BD-2 also has chemotactic activity^17^ which may increase recruitment of HIV target cells to mucosal sites thereby facilitating viral transmission, which may explain why higher BD2 was associated with risk of HIV acquisition.^7^ Other studies (reviewed in^18 19^) suggest that the role of both alpha and beta defensins in viral infection may be more complex in vivo and may be altered by other factors in the female genital tract such as the microbiome and STIs. It is possible that high cervical BD-2 is a consequence of an underlying undiagnosed microbiome shift or asymptomatic STI.

Systemic immunity can be functionally distinct from mucosal immunity in the female genital tract^20 21^ and measuring markers in both compartments has provided distinct biomarkers of HIV acquisition risk.^7 22^ Prior to our study, there were no published data on systemic immunity preceding HSV-2 acquisition. We report that high systemic sCD14 and IL-6, both alone and combined, predict HSV-2 acquisition. Interestingly, the effect of higher sCD14 on HSV-2 risk was offset by concomitant higher cervical IL-6 and IL-1β. IL-6 is a pleiotropic cytokine implicated in the pathogenesis of various infectious and non-infectious diseases.^23^ IL-6 is part of the antiviral stress response and thus its mucosal upregulation may be a sign of more robust antiviral barrier. Targeting IL-6 systemically constitutes a treatment strategy for inflammation-mediated diseases including diabetes, obesity and rheumatoid arthritis.^24^ Systemically altered IL-6 may play a role in malnourishment pathophysiology,^25^ and its levels in the circulation are also controlled by genetic polymorphisms. Therefore, it is possible that high systemic IL-6 may not directly affect HSV-2 pathogenesis at the mucosal site but could be indicative of an underlying unmeasured exposure, disease or condition that may independently increase susceptibility to HSV-2. sCD14 is a marker for monocyte activation and microbial translocation from the mucosal surface to the systemic circulation and may be a sign of mucosal damage.^26^ Bacterial endotoxins and inflammatory cytokines such as IL-6 can induce the release of sCD14 in the circulation.^27^ Considering this biological link between IL-6 and sCD14, it is not unexpected that their serum levels show the same direction of association.

The combination of systemic CRP↓ and cervical BD-2↑, associated with HSV-2 risk in this study, was also found to precede HIV-1 acquisition.^7^ CRP is primarily produced in the liver but also by lymphocytes and other cell types and low CRP may interfere with its important part in innate antiviral immune responses including completement activation.^28^

We have further demonstrated suppressed innate immunity with differences between women with incident and established HSV-2 infections. Women with incident infections had lower levels of seven cervical markers (SLPI, IL-1RA, IL-1β, IL-6, IL-8, MIP-3α and VEGF) while women with established infections had lower levels of four of these seven markers and lower cervical BD-2 compared with HSV-2 negative visits. In a direct comparison with established infections, women with incident infections had lower levels of cervical IL-1β and MIP-3α (an anti-HIV microbicide^29^) and lower systemic IL-6. The lower serum IL-6 previously found predictive of HIV-1 acquisition^7^ offered one plausible biological link between higher risk for HIV-1 with incident than established HSV-2 infections. The overall immunosuppressed state may also contribute to higher HIV susceptibility among both incident and prevalent infections compared with HSV-2 negative women.

Our study has several important strengths. We followed over a thousand women with roughly equal-sized groups of women with incident or established HSV-2 infections and women remaining HSV-2 negative. We were able to analyse large numbers of participant visits with matched cervical and serum specimens thus allowing us to examine the relative and combined contributions of immune factors from each of these anatomical compartments. HSV-2 infection was measured at each 12-week visit providing us with accurate information about the timing of incident infections and accurately identifying the three HSV-status groups. For the HSV-2 infection status model, we adjusted for relevant confounders thus strengthening associations between the biomarkers and incident versus established infections. In the model predictive of HSV-2 acquisition, we did not control for behavioural and demographic factors as evidence^8^ from this cohort suggested that the immune markers were more proximate to HSV-2 infection and thus control for antecedent variables was inappropriate. In addition, all biomarkers were measured at the same accredited laboratory with methods previously validated for technical accuracy and clinical content.^11^ A limitation of the dataset is that not all women contributed both HSV incident and established visits, precluding comparisons where all women served as their own controls. Future validation studies should be conducted in ethnically and racially diverse populations and larger groups of pregnant women.

## Conclusion

This study furthers understanding of HSV-2 acquisition and the complex link between HSV-2 and HIV-1 by providing clinical evidence for divergent and shared mechanisms of vulnerability to viral infection. The identified molecular predictors of HSV-2 risk provide targets and clinical safety endpoints for the development of preventive products. The discovered systemic and mucosal immunity patterns distinguishing incident from established HSV-2 infection should be studied in relationship to viral load and shedding.

10.1136/sextrans-2022-055458.supp2 Abstract translation . This web only file has been produced by the BMJ Publishing Group from an electronic file supplied by the author(s) and has not been edited for content.

## Data Availability

Data are available on reasonable request. All data will be made available on reasonable request to be submitted to the corresponding author.
