# Supplementary material for: Cervical and systemic innate immunity predictors of HIV risk linked to genital herpes acquisition and time from HSV-2 seroconversion
Source: Sex Transm Infect. 2022 Sep 14;99(5):311–6. doi: 10.1136/sextrans-2022-055458 (PMC10011014; doi:10.1136/sextrans-2022-055458)
Supplement: Supplementary data [file sextrans-2022-055458supp001.pdf]

**Supplementary Table 1.** Correlation between cervical and serum biomarkers as continuous variables adjusted by repeated measures among participants. The correlation matrix shows estimated Spearman rank-order correlation coefficients (top of the grid) with p-vales (bottom of the grid) calculated by t-distributions. The coefficient indicates the degree of correlation which is ranked weak if a coefficient is between -0.2 and 0.2 and strong if <-0.7, >0.7). The p-value indicates the chance of zero correlation coefficient between two biomarkers.

| | Cervical Biomarkers | | | | | | | | | | Serum Biomarkers | | | |
|---|---|---|---|---|---|---|---|---|---|---|---|---|---|---|
| | BD2 | SLPI | IL-1RA | IL-1β | IL-6 | IL-8 | MIP-3α | RANTES | ICAM-1 | VEGF | sCD14 | CRP | IL-6 | IL-7 |
| BD2 | | 0.0272 | 0.25733 | 0.19236 | 0.13071 | 0.2556 | 0.22339 | -0.205 | -0.1403 | 0.15259 | 0.04608 | -0.0105 | -0.0473 | -0.004 |
| | | 0.1881 | <.0001 | <.0001 | <.0001 | <.0001 | <.0001 | <.0001 | <.0001 | <.0001 | 0.0257 | 0.6104 | 0.0221 | 0.8479 |
| SLPI | | | 0.23074 | 0.30286 | 0.46831 | 0.44998 | 0.56425 | 0.0763 | 0.2796 | 0.39932 | -0.1711 | 0.13425 | -0.1447 | -0.0591 |
| | | | <.0001 | <.0001 | <.0001 | <.0001 | <.0001 | 0.0002 | <.0001 | <.0001 | <.0001 | <.0001 | <.0001 | 0.0042 |
| IL-1RA | | | | 0.34591 | 0.29607 | 0.24634 | 0.36045 | -0.1206 | 0.14265 | 0.39848 | 0.00271 | 0.00915 | -0.0053 | 0.04161 |
| | | | | <.0001 | <.0001 | <.0001 | <.0001 | <.0001 | <.0001 | <.0001 | 0.8958 | 0.6581 | 0.7993 | 0.044 |
| IL-1β | | | | | 0.52279 | 0.62527 | 0.46096 | 0.01093 | 0.22328 | 0.5375 | -0.1061 | 0.09958 | -0.0095 | 0.02462 |
| | | | | | <.0001 | <.0001 | <.0001 | 0.5968 | <.0001 | <.0001 | <.0001 | <.0001 | 0.6461 | 0.2334 |
| IL-6 | | | | | | 0.69766 | 0.64149 | 0.10954 | 0.33106 | 0.62496 | -0.1362 | 0.13037 | -0.1042 | -0.0417 |
| | | | | | | <.0001 | <.0001 | <.0001 | <.0001 | <.0001 | <.0001 | <.0001 | <.0001 | 0.0434 |
| IL-8 | | | | | | | 0.62738 | 0.04736 | 0.20064 | 0.65417 | -0.1188 | 0.12726 | -0.0727 | -0.0024 |
| | | | | | | | <.0001 | 0.0218 | <.0001 | <.0001 | <.0001 | <.0001 | 0.0004 | 0.9077 |
| MIP-3α | | | | | | | | 0.11866 | 0.30056 | 0.49819 | -0.109 | 0.11568 | -0.111 | -0.0135 |
| | | | | | | | | <.0001 | <.0001 | <.0001 | <.0001 | <.0001 | <.0001 | 0.5149 |
| RANTES | | | | | | | | | 0.53644 | 0.00471 | 0.00933 | -0.0402 | -0.0707 | -0.0986 |
| | | | | | | | | | <.0001 | 0.8197 | 0.6517 | 0.0517 | 0.0006 | <.0001 |
| ICAM-1 | | | | | | | | | | 0.23479 | -0.0613 | 0.01135 | -0.0217 | -0.037 |
| | | | | | | | | | | <.0001 | 0.003 | 0.5829 | 0.2946 | 0.0732 |
| VEGF | | | | | | | | | | | -0.0831 | 0.10944 | -0.0499 | 0.00777 |
| | | | | | | | | | | | <.0001 | <.0001 | 0.0156 | 0.7069 |
| sCD14 | | | | | | | | | | | | -0.0152 | 0.16132 | 0.09685 |
| | | | | | | | | | | | | 0.4613 | <.0001 | <.0001 |
| CRP | | | | | | | | | | | | | 0.15961 | 0.09542 |
| | | | | | | | | | | | | | <.0001 | <.0001 |
| IL-6 | | | | | | | | | | | | | | 0.20992 |
| | | | | | | | | | | | | | | <.0001 |
| IL-7 | | | | | | | | | | | | | | |

Supplementary **Table 2.** Characteristics of participant visits by HSV-2 status among HIV negative visits

| Characteristics | HSV-2 negative[1] visits (n=1505) n (%) | HSV-2 incident[2] visits (n=633) n (%) | HSV-2 established[3] visits (n=978) n (%) | Total (n=3116) n (%) | P-value |
|---|---|---|---|---|---|
| **Site** | | | | | |
| Uganda | 518 (34.42) | 318 (50.24) | 294 (30.06) | 1130 (36.26) | |
| Zimbabwe | 987 (65.58) | 315 (49.76) | 684 (69.94) | 1986 (63.74) | **<.001** |
| **Age at screening** | | | | | |
| 18-24 | 989 (65.71) | 383 (60.51) | 469 (47.96) | 1841 (59.08) | |
| 25+ | 516 (34.29) | 250 (39.49) | 509 (52.04) | 1275 (40.92) | **<.001** |
| **HC group** | | | | | |
| COC | 535 (35.55) | 230 (36.33) | 398 (40.74) | 1163 (37.34) | |
| DMPA | 545 (36.21) | 179 (28.28) | 280 (28.66) | 1004 (32.23) | |
| NH | 425 (28.24) | 224 (35.39) | 299 (30.6) | 948 (30.43) | **<.001** |
| **Currently pregnant** | 111 (7.4) | 42 (6.68) | 99 (10.16) | 252 (8.12) | **<.001** |
| **Currently Breastfeeding** | 243 (16.15) | 98 (15.51) | 118 (12.07) | 459 (14.74) | **<.001** |
| **2+ sexual partners** | 29 (1.93) | 11 (1.74) | 12 (1.23) | 52 (1.67) | **<.001** |
| **Current smoking** | 3 (0.2) | 1 (0.16) | 4 (0.41) | 8 (0.26) | **<.001** |
| **# unprotected acts** | | | | | |
| 15+ | 339 (22.52) | 133 (21.01) | 234 (23.93) | 706 (22.66) | |
| 8-14 | 390 (25.91) | 181 (28.59) | 237 (24.23) | 808 (25.93) | |
| 1-7 | 401 (26.64) | 131 (20.7) | 210 (21.47) | 742 (23.81) | |
| 0 or no sex act | 375 (24.92) | 188 (29.7) | 297 (30.37) | 860 (27.6) | **<.001** |
| **Any RTI/STI** | 593 (40.9) | 633 (100) | 978 (100) | 2204 (72) | **<.001** |
| BV (Nugent score 7-10) | 391 (27.08) | 166 (27.35) | 272 (29.76) | 829 (27.96) | **<.001** |
| Candidiasis | 169 (11.3) | 66 (10.59) | 100 (10.49) | 335 (10.91) | **<.001** |
| Chlamydia | 32 (2.15) | 19 (3.09) | 10 (1.06) | 61 (2) | **<.001** |
| Gonorrhea | 33 (2.22) | 19 (3.09) | 13 (1.37) | 65 (2.13) | **<.001** |
| Trichomonas | 42 (2.83) | 22 (3.57) | 17 (1.81) | 81 (2.66) | **<.001** |

[1] HSV-2 negative visits were contributed by 382 women remaining HSV-2 negative (1157 visits) and 334 women that HSV-2 seroconverted during the study contributed (348 negative visits occurring at least 3 months before the HSV-2 seroconversion visit).

[2]The 633 visits defined as 'HSV-2 incident visits' were contributed by 334 women who passed inclusion criteria and included all available visits by these women that occurred at and <6 months (180 days) after the first HSV-2 positive test.

[3]The 978 visits defined as 'HSV-2 established' were contributed by: 1) 303 women who were HSV-2 positive at enrollment (528 visits available at least 6 months from the enrollment visit), and 2) 334 women who acquired HSV-2 during the study (450 visits available at ≥ 6 months from the HSV-2 seroconversion visit).

**Supplementary Table 3**. Estimated odds ratios (OR) of HSV-2 acquisition for levels of concomitantly imbalanced systemic and cervical Immunity measured at the HSV-2 negative visits from individuals remaining HSV-2 negative and the HSV-2 seroconverters at the visit prior to the incident visit.

| Systemic + Cervical Biomarker Groups | #↑↓[1] / #Sample used[2] | OR[3] (95% CI) | P-value |
|---|---|---|---|
| ***Concomitant serum sCD14 ↑ + cervical ↑↓*** | | | |
| IL-1β↑, IL-6↑ | 48 / 703 | 0.39 (0.14, 1.11) | 0.077 |
| IL-8↑, MIP-3α↑ | 52 / 703 | 0.68 (0.30, 1.55) | 0.360 |
| VEGF↓, ICAM-1↓ | 47 / 703 | **2.02 (1.04, 3.90)** | **0.037** |
| SLPI↓ | 84 / 703 | **1.85 (1.09, 3.12)** | **0.022** |
| BD-2↑ | 72 / 702 | **2.18 (1.26, 3.75)** | **0.005** |
| IL-1RA↓ | 67 / 703 | 1.34 (0.72, 2.46) | 0.354 |
| RANTES↑ | 87 / 703 | 1.30 (0.75, 2.25) | 0.351 |
| ***Concomitant serum CRP ↓ + cervical ↑↓*** | | | |
| IL-1β↑, IL-6↑ | 114 / 703 | 0.75 (0.43, 1.32) | 0.322 |
| IL-8↑, MIP-3α↑ | 125 / 703 | 1.15 (0.71, 1.88) | 0.568 |
| VEGF↓, ICAM-1↓ | 96 / 703 | 0.96 (0.55, 1.69) | 0.892 |
| SLPI↓ | 169 / 703 | 1.36 (0.89, 2.10) | 0.156 |
| BD-2↑ | 168 / 702 | **1.82 (1.20, 2.76)** | **0.005** |
| IL-1RA↓ | 179 / 703 | 1.18 (0.77, 1.82) | 0.446 |
| RANTES↑ | 207 / 703 | 1.21 (0.80, 1.83) | 0.357 |
| ***Concomitant serum IL6 ↑ + cervical ↑↓*** | | | |
| IL-1β↑, IL-6↑ | 121 / 703 | **0.44 (0.24, 0.83)** | **0.012** |
| IL-8↑, MIP-3α↑ | 132 / 703 | 0.88 (0.53, 1.45) | 0.612 |
| VEGF↓, ICAM-1↓ | 89 / 703 | **1.94 (1.16, 3.23)** | **0.011** |
| SLPI↓ | 164 / 703 | **1.65 (1.08, 2.53)** | **0.020** |
| BD-2↑ | 156 / 702 | 1.41 (0.91, 2.18) | 0.125 |
| IL-1RA↓ | 156 / 703 | 1.41 (0.91, 2.19) | 0.123 |
| RANTES↑ | 174 / 703 | 1.07 (0.69, 1.66) | 0.766 |

[1] ↑ indicates activated (levels above median) and ↓ indicates suppressed (levels below median) immunity, with the exception of sCD14 which is activated if its concentration is above top quartile, for all HIV-1 and HSV-2 negative visits.

[2]HSV-2 negative visits from individuals remaining HSV-2 negative and HSV-2 seroconverters at the visit prior to the incident visit.

[3]Odds ratios (OR) and 95% confidence intervals (CI) estimated likelihood of HSV-2 seroconversion with biomarker levels activated/suppressed as indicated by arrows assessed concomitantly.
