# Supplementary material for: Cervical and systemic innate immunity predictors of HIV risk linked to genital herpes acquisition and time from HSV-2 seroconversion
Source: Sex Transm Infect. 2022 Sep 14;99(5):311–6. doi: 10.1136/sextrans-2022-055458 (PMC10011014; doi:10.1136/sextrans-2022-055458)
Supplement: Abstract translation [file sextrans-2022-055458supp002.pdf]

Маркери на маточно-цервикален и системен вроден имунитет предразполагащ към HIV-1 свързани с риска от придобиване на генитален херпес и с времетраенето след HSV-2 сероконверсия

Резюме

Цели: Да се изследват маркери на вродения имунитет, които предричат риск от HIV-1, като прекурсори на генитален херпес и като биологична основа за епидемиологично установеното предразположение към HIV-1 при жени заразени с HSV-2.

Методи: Ние анализирахме серийни HIV-отрицателни серумни и маточно-цервикални проби от 1019 жени преди и след придобиване на генитален херпес. В тях измерихме биомаркери на възпаление и имунно активиране, по-рано свързани с риск от заразяване с HIV-1. Концентрациите им бяха трансформирани чрез Box-Cox и коефициенти на риск от придобиване на HSV-2 бяха изчислени въз основа на вероятността от маркерни нива над медианата или в горния 25 процентов интервал спрямо всички HSV-2 отрицателни проби. Двувариантен анализ определи вероятността от придобиване на HSV-2 чрез нивата на биомаркерите преди инфекцията. Линейни статистически модели със смесени ефекти бяха приложени за установяване на разлики свързани с HSV-2 статуса, дефиниран като отрицателен, първа инфекция в първите 6 месеца на сероконверсия или статус на инфекция след шестия месец от сероконверсия.

Резултати: В цервикалните секрети два рискови биомаркера на HIV-1 (нисък SLPI и висок BD-2) предсказаха също придобиването на HSV-2. В допълнение, придобиването на HSV-2 беше свързано с ниски нива на IL-1β, IL-6, IL-8, MIP-3α, ICAM-1 и VEGF. Системно-имунни предиктори на HSV-2 инфекция бяха високите серумни нива на sCD14 и IL-6, свързани с най-висок риск при едновременно повишаване (OR=2,23, 1,49-3,35). Системно-мукозно съпътстващите предиктори на риск от придобиване на HSV-2 бяха: 1) висок серумен sCD14 комбиниран с ниски цервикални нива на SLPI, VEGF и ICAM-1 или с висок BD-2; 2) висок серумен IL-6 комбиниран с ниски цервикални нива на VEGF и ICAM-1, SLPI, или IL-1β и IL-6 и 3) нисък серумен CRP комбиниран с висок цервикален BD-2 (единствената комбинация, която също предсказва придобиването на HIV-1). Повечето цервикални биомаркери бяха понижени след придобиване на HSV-2 в сравнение с HSV-2 отрицателния статус, като пробите по-близо след първо заразяване (в 6-месечен

интервал) показаха по-голям брой подтиснати цервикални биомаркери и по-ниски серумни нива на IL-6 в сравнение с по-късните периоди на инфекция.

Заключения: Комбинацията от системно възпаление и маточно-цервикална имунна супресия предлазполага към HSV-2. Трайно подтиснатият вроден имунитет в първите 6 месеца след HSV-2 инфекция може да допринесе за повишена податливост към HIV-1 инфекция.

Ключови послания

Какво вече е известно по тази тема: Гениталният херпес е разпространен рисков фактор за заразяване с ХИВ, но не е известно дали едни и същи белези на лигавичния и/или системния вроден имунитет предхождат и предлазполагат към двете вирусни инфекции.

С какво допринася това проучване: Това проучване идентифицира за първи път както общи, така и диференциални вродено-имунни предиктори на HSV-2 и HIV-1 инфекциии. То е първото, което показва разлики както в медиаторите на вродения имунитет на шийката на матката, така и в системния вроден имунитет, които могат да лежат в основата на по-високия риск от заразяване с ХИВ вързан със скорошна първа инфекция с HSV-2 спрямо последващ по-късен период след серконверсия.

Как това проучване може да повлияе на науката, клиничната практика или политиката на здравеопазване: Чрез идентифициране на молекулярни предиктори на риска от HSV-2, това проучване предоставя лекарствени мишени и лабораторни критерии за клинична безопасност необходими за разработването на профилактични продукти.

Автор-кореспондент: Проф. Д-р Райна Фичорова rfichorova@bwh.harvard.edu
